# Efficacy and safety of Shenkang injection in the treatment of chronic renal failure: A protocol of a randomized controlled trial

**DOI:** 10.1097/MD.0000000000027748

**Published:** 2021-12-03

**Authors:** JuXiang Mei, LingLing Yang, Deqin Wang, HaiXia Wang

**Affiliations:** Hai’an Hospital Affiliated to Nantong Medical College, Nantong, Jiangsu, China.

**Keywords:** chronic renal failure, randomized controlled trial, Shenkang injection

## Abstract

**Background:**

Chronic renal failure (CRF) is the final outcome of the development of multiple kidney diseases, and there is no effective method at home and abroad. Traditional Chinese medicine is found to play a major role in the treatment of the non-replacement stage of CRF. Shenkang injection can not only nourish the kidney, but also promote blood circulation and remove blood stasis, which is suitable for the treatment of CRF. This study aims to explore the efficacy and safety of Shenkang injection for CRF and provide evidence for clinical practice.

**Methods:**

This was a prospective randomized controlled trial. One hundred four patients with CRF were randomly divided into treatment groups and control groups according to 1:1, with 52 patients in each group. The control group received basic treatment of western medicine and the treatment group was given Shenkang injection intravenously on the basis of control group. Both groups were given standard treatment for 4 weeks with concurrent follow-up for 1 month. The outcome indicators included: total efficiency, symptom scores, creatinine clearance rate, serum creatinine, blood urea nitrogen, CystatinC, liver function, blood routine, urine routine, incidence of adverse reactions, etc. Data analysis was performed using SPSS 25.0 software.

**Discussion:**

This study will evaluate the efficacy and safety of Shenkang injection for CRF, and the results of this trial will provide clinical evidence for the treatment of CRF.

**Trial registration:**

OSF Registration number: DOI 10.17605/OSF.IO/K9C5T.

## 1 Introduction

Chronic renal failure (CRF) is mainly characterized by renal fibrosis.^[1]^ It is a common urinary system disease in the elderly population,^[2]^ often involved in the cardiovascular, blood, digestive tract, and other major systems.^[3]^ Clinical treatment of CRF is difficult, and the poor prognosis and high mortality seriously affect the quality of life of patients.^[4]^ In the United States, about 745,000 patients have had end-stage kidney disease^[5]^; In China, more than 300,000 patients underwent dialysis by the end of 2014,^[6]^ and is still growing at 7% a year.^[7]^ CRF has become a global public health problem. Western medicine treatment is mainly divided into conservative treatment and renal replacement treatment, but it is still not ideal in reducing complications, preventing the deterioration process, and the long-term survival rate.^[8]^ Therefore, how to prevent its chronic deterioration and protect kidney function has been the focus of research in the field of kidney disease. Clinical studies have found that traditional Chinese medicine (TCM) has shown its unique efficacy in delaying CRF.

The treatment of CRF in TCM focuses on tonifying spleen and kidney and promoting blood circulation, detoxification, and relieving turbidity.^[9]^ Shenkang injection is a compound injection of TCM. The main ingredients are dahuang (*Radix et Rhizoma Rhei*), danshen (*Radix Salviae Miltiorrhiae*), honghua (*Flos Carthami*), huangqi (*Radix Astragali*), which have the effect of calming the adverse-rising energy and relieving turbidity, replenishing qi and promoting blood circulation, and benefiting visceral dampness. Modern pharmacological studies have found that Shenkang injection can act by regulating the transforming growth factor-β1/Smad3 protein^[10]^ and mitogen-activated protein kinase, which inhibits renal fibrosis and oxidative stress response,^[11]^ has anti-inflammatory,^[12]^ anti-renal fibrosis,^[13,14]^ and improves coagulation function.^[15]^ Traditional Chinese medicine has significant advantages in improving patient symptoms and renal function,^[16,17]^ and has few adverse reactions. Intravenous medication has obvious effects with few side effects.^[18]^

Therefore, we plan to evaluate the efficacy and safety of Shenkang injection for CRF through this randomized controlled trial.

## 2 Methods

### 2.1 Study design

**Figure 1:**
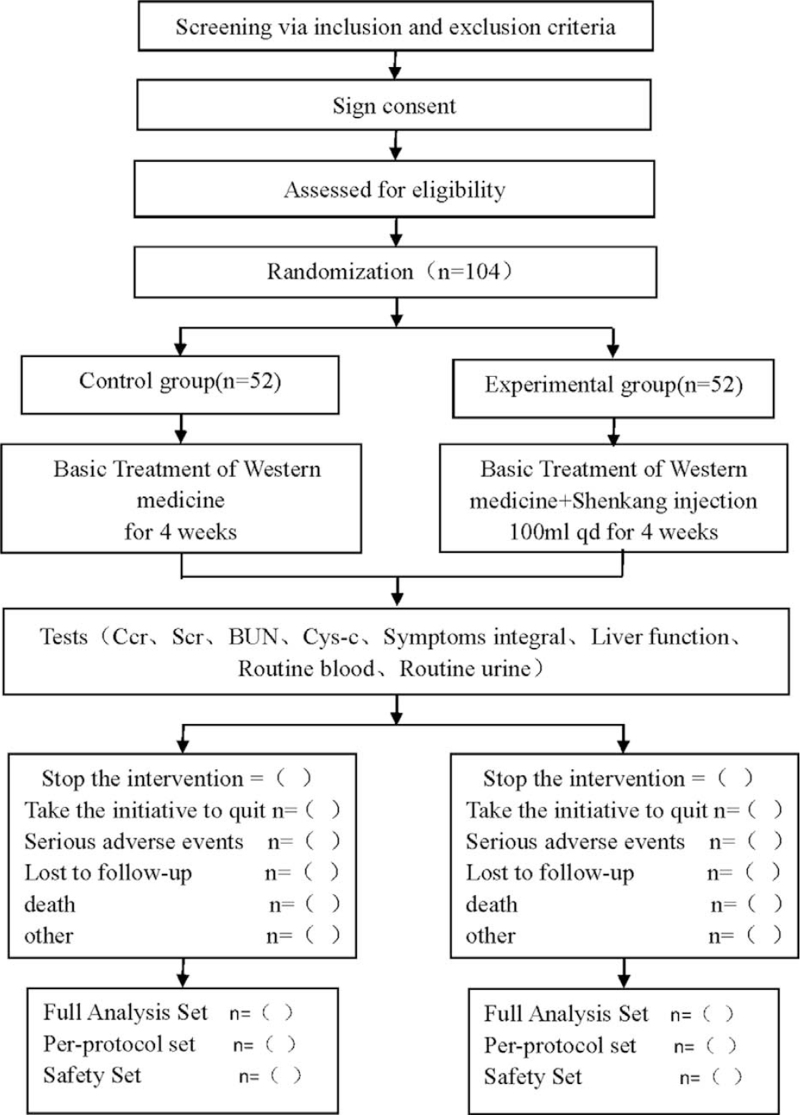
Flow diagram. Bun = blood urea nitrogen, Ccr = creatinine clearance rate, Cys-c = CystatinC, Scr = serum creatinine.

This was a prospective randomized controlled trial, to study the efficacy and safety of Shenkang injection for CRF. This study protocol followed the latest Consolidated Standards of Reporting Trials (CONSORT 2017) (see Fig. 1 for the flow chart), and Standard Protocol Items: Recommendations for Interventional Trials (SPIRIT) 2013 statement.

### 2.2 Ethics and registration

The study protocol was in accordance with the *Declaration of Helsinki* and was approved by the ethics committee of our hospital. This experiment has been registered in the open science framework (OSF) with the registration number: DOI 10.17605/OSF.IO/K9C5T. Informed consent was required for all patients, and patients who met the inclusion and standard criteria were then randomly assigned.

### 2.3 Patients

Diagnostic criteria: the definition and staging of CKD^[19]^ in the *Clinical Practice Guidelines for CKD* issued by Kidney Disease Outcome Quality Initiative in 2002 was adopted to classify CKD into stages 1 to 5.

Inclusion criteria: metting the diagnostic criteria for CRF, chronic kidney disease stage 3 to 5; age ≥18 years and ≤80 years; no dialysis treatment was performed; voluntarily accepted corresponding treatment and cooperate; the informed consent was signed.

Exclusion criteria: those combined with other serious organ dysfunction; those suffering from other serious diseases such as malignant tumors; those with severe mental illness; those allergic to the test drugs; those who have participated in or are participating in other clinical trials in nearly 1 month; those unable to understand the study protocol or unwilling to attend participants after explanation.

Excluding criteria: serious complications and serious adverse events that were not suitable for the next test; poor compliance, affecting the outcome judgment; patients whose disease progresses rapidly during treatment and need to change treatment plan; for any reason, the subject asked to quit the study.

### 2.4 Sample size

Since the primary efficacy index of this study was the overall efficiency, sample size estimates were therefore performed based on the total efficient pre-experiments. According to the preclinical preexperimental findings, the total efficiency of Shenkang injection combined with basic western medicine treatment was 93.9%, and the total efficiency of basic western medicine treatment was 75%. PASS15.0 was used for sample size estimation, and optimization design was adopted, where α = 0.05, β = 0.2, test efficiency = 0.8, the number of test groups: the number of cases in the control group = 1:1, and the boundary value = –0.1. According to the software calculation, the total sample size of the 2 groups was 92 cases. Considering the clinical shedding rate of about 10%, a total of 104 cases were finally included, 52 cases in each group.

### 2.5 Random and assignment hidden

By completely randomization, 104 patients were included, 104 patients were sequentially numbered 1 to 104; Use Excel2013 software to input =RANDBETWEEN (1,1000) in the corresponding cell on the right of each number. Click enter to automatically generate random numbers, and then arrange the random numbers in ascending order. Set the first 52 as treatment group and the last 52 as control group. The grouping of each patient was put in an opaque envelope in advance, and the envelope was opened after the patient met the inclusion and exclusion criteria and voluntarily agreed to participate in the study, and the corresponding treatment plan was implemented according to the group of the envelope.

### 2.6 Interventions

The 2 groups received the same routine care, avoiding tobacco, alcohol, and stimulating food during the study, avoiding staying up late and were payed attention to adverse reactions. All intervention modalities were recorded in detail and used for the final statistical analysis. The efficacy assessors did not know the study group protocol, and the data statistics did not participate in the study design and implementation. The health of each patient was assessed before and after treatment, including observation indicators, and all patients were followed up by telephone.

- Control group: such as low salt, low fat, low quality protein diet, control blood pressure, blood sugar, correct water, and electrolyte disorders. Patients combined with hypertensive were given oral antihypertensive drugs, patients combined with diabetes patients were treated with insulin, and patients combined with anemia were given subcutaneous injection of erythropoietin to correct renal anemia. The treatment lasted for 4 weeks.
- Treatment group: on the basis of the control group, Shenkang injection (Xi‘an Shiji Shengkang Pharmaceutical Co., LTD., National Drug Approval Z10040110,20 mL/branch)100 mL +10% glucose 250 mL, once a day, lasted for 4 weeks.

### 2.7 Outcomes

#### 2.7.1 Observational index

- Symptoms scores: including fatigue, reduced appetite, fatigue, waist and knee weakness, nausea, vomiting, limb sleepiness, limb numbness. The score grade was divided into 4 grades: no, light, medium and heavy. They were recorded once before and after 4 weeks of medication.
- Laboratory indexes: Creatinine clearance rate (Ccr), serum creatinine (Scr), blood urea nitrogen, CystatinC.

The records were recorded once before medication, 2 weeks after medication and 4 weeks after medication.

#### 2.7.2 Efficacy indicators

Main efficacy indicators: total efficiency: refer to the *Guidelines for Clinical Research of New Chinese Medicine Drugs*,^[20]^ total effective rate = (effective number + effective number)/total number of patients ×100%. significant effect: clinical symptom score decreased ≥60%, Ccr increased ≥20% or Scr decreased ≥20%; effective: 30%≤ clinical symptom score decreased <60%, 10%≤ Ccr increased <20% or 10%≤ Scr decreased <20%; stable: clinical symptom score decreased by <30%, Ccr increased by <10% or Scr decreased by <10%; invalid: the clinical symptoms were not improved or aggravated, the clearance rate of Ccr decreased, and the Scr increased.

Secondary efficacy indicators: changes of Ccr, Scr, blood urea nitrogen, CystatinC before and after treatment.

#### 2.7.3 Safety indicators

Liver function, blood routine, urine routine, recorded once before and 4 weeks after medication.

#### 2.7.4 Incidence of adverse events

Including the frequency of any uncomfortable symptoms during treatment.

### 2.8 Study quality control

Safety monitoring was given for each participant throughout the trial. The occurrence of all adverse events would be reported to the Ethics committee. We set up the Data and Safety Monitoring Committee Board (DSMB). Members of the Data and Safety Monitoring Committee Board included physicians, trial method specialists, clinical pharmacists, statistical experts, and members of the ethics committee, who would conduct risk assessment and safety analysis procedures according to termination conditions.

### 2.9 Statistical analysis plan

Excel were used to establish the database, and the efficacy indicators were analyzed by full analysis set and per-protocol set. Safety data set was used for Safety analysis.

SPSS25.0 statistical analysis software was used for data analysis in this study. If measurement data were in line with normal distribution, the results were represented by Mean±standard deviation ($\bar{x}\pm \text{S}$). Paired sample *T* test was used within groups, and independent sample *T* test was used between groups. For those not conforming to normality, the results were represented by quartiles and nonparametric test. Chi-square test was used for counting data. The incidence of adverse events was compared by chi-square test. *P *<* *.05 was statistically significant.

## 3 Discussion

CRF is a pathological disease characterized by the destruction of renal tissue structure and the loss of renal function due to the excessive accumulation of extracellular matrix due to the excessive expression of some cytokines, dysregulation of cell metabolism and excessive proliferation caused by various pathogenic factors.^[21]^ Studies have shown that the occurrence and development of CRF is closely related to the overactivation of renin-angiotensin system, which mediates cell growth and proliferation to promote fibrosis. With the aggravation of renal fibrosis, the course of CRF progresses.^[1,22]^ Basic treatment in western medicine is mainly to delay the progression of CRF by controlling risk factors, treating the primary disease and symptomatic treatment, but the improvement of symptoms, long-term life and the treatment effect of complications are still not satisfactory. In recent years, TCM agents have been widely used in CRF patients and achieved remarkable results, providing news for clinical treatment of patients with CRF. The treatment of CRF with integrated traditional Chinese medicine and western medicine has become a trend of clinical treatment.

Shenkang injection contains dahuang (*Radix et Rhizoma Rhei*), danshen (*Radix Salviae Miltiorrhiae*), honghua (*Flos Carthami*), and huangqi (*Radix Astragali*). Modern studies have found that dahuang (*Radix et Rhizoma Rhei*) can improve kidney function and improve pathological kidney damage,^[23]^ reduce the LPS-induced inflammatory macrophage response,^[24]^ reduce renal fibrosis and delay renal failure^[25]^; huangqi (*Radix Astragali*) can protect glomerular mesangial cells and reduce proteinuria,^[26]^ promote water and sodium excretion, and protect the kidney^[27]^; danshen (*Radix Salviae Miltiorrhiae*) has anti-inflammatory, anticoagulant, and antioxidant properties^[28]^ and anti-anemia effect,^[29]^ can effectively improve the microcirculation in CRF patients.

Since there are no standard large sample clinical studies to evaluate the efficacy and safety of Shenkang injection in patients with CRF, we propose to evaluate its efficacy through this prospective randomized controlled study.

This study also has several limitations: with a short follow-up time, we were unable to understand the impact of long-term efficacy and, therefore, we may extend the follow-up time if necessary.

## Author contributions

**Data collection:** JuXiang Mei and LingLing Yang.

**Funding support:** HaiXia Wang.

**Investigation:** JuXiang Mei and Deqin Wang.

**Resources:** LingLing Yang and Deqin Wang.

**Software operating:** HaiXia Wang and Deqin Wang.

**Supervision:** Deqin Wang and HaiXia Wang.

**Writing - original draft:** HaiXia Wang and LingLing Yang.

**Writing - review & editing:** JuXiang Mei and HaiXia Wang.
